# Supplementary material for: Genetic Features of Mycobacterium avium subsp. paratuberculosis Strains Circulating in the West of France Deciphered by Whole-Genome Sequencing
Source: Microbiol Spectr. 2022 Nov 29;10(6):e03392-22. doi: 10.1128/spectrum.03392-22 (PMC9769990; doi:10.1128/spectrum.03392-22)
Supplement: Supplemental file 1 — Supplemental material. Download spectrum.03392-22-s0001.pdf, PDF file, 0.3 MB [file spectrum.03392-22-s0001.pdf]

Supplementary Figure S1: Mauve alignment of K-10 (top) with the four newly assembled French genomes (PICSAR51, PICSAR68, PICSAR77, PICSAR142 respectively) showing remarkable genomic stability of the *Map* strains. The colored boxes represent homologous regions present in each genome. Blocks below the centerline indicate regions with inverse orientation. There are no regions outside the blocks, suggesting complete homology between the genomes. The scale is in base pairs. Only one large inversion was shared among the four strains with respect to the K-10 reference genome.

Supplementary Figure S2: Non-random distribution of MLVA profiles among the 3 phylogenetic clades. Bar plot of strains versus clades representing the proportion of strains from each clade assigned to the two major MLVA profiles. Note the predominance of INMV1 strains in clade A compared to the predominance of INMV2 strains in clade B.

Supplementary Figure S3: Geographic locations of *Map* strain profiles. Shown is the enlarged section of western France taken from the inset at the top left. The colors correspond to the MLVA profiles of the isolates collected in the herd. Circle sizes represent the number of samples collected in the herd (size scale in the lower left). The map was produced using MicroReact (v202) (76).

Supplementary Table S1: Metadata for each dataset used in this study.

Supplementary Table S2: Non-core genes found in the pangenome analysis of the 153 *Ma*p genomes.

Supplementary Table S3: Clade specific SNPs and their functional annotation. The positions are colored according to the clade they discriminate. The genome coordinates in column A are from PICSAR77. Strains containing SNPs relative to PICSAR77 are listed across in row 1.

Supplementary Table S4: Details on animals grouped by herds with associated strains. Rows highlight in yellow are cases described in the results.

Supplementary Table S5: Association index of traits with the ML phylogeny. Redundancies in the phylogeny were removed for this analysis.

Supplementary Table S6: Data and genotypes obtained from the variant call regarding the non-synonymous mutation in the gyrB gene at position 1124. The functional annotation generated by snpEff and the country of origin of the strains were included.

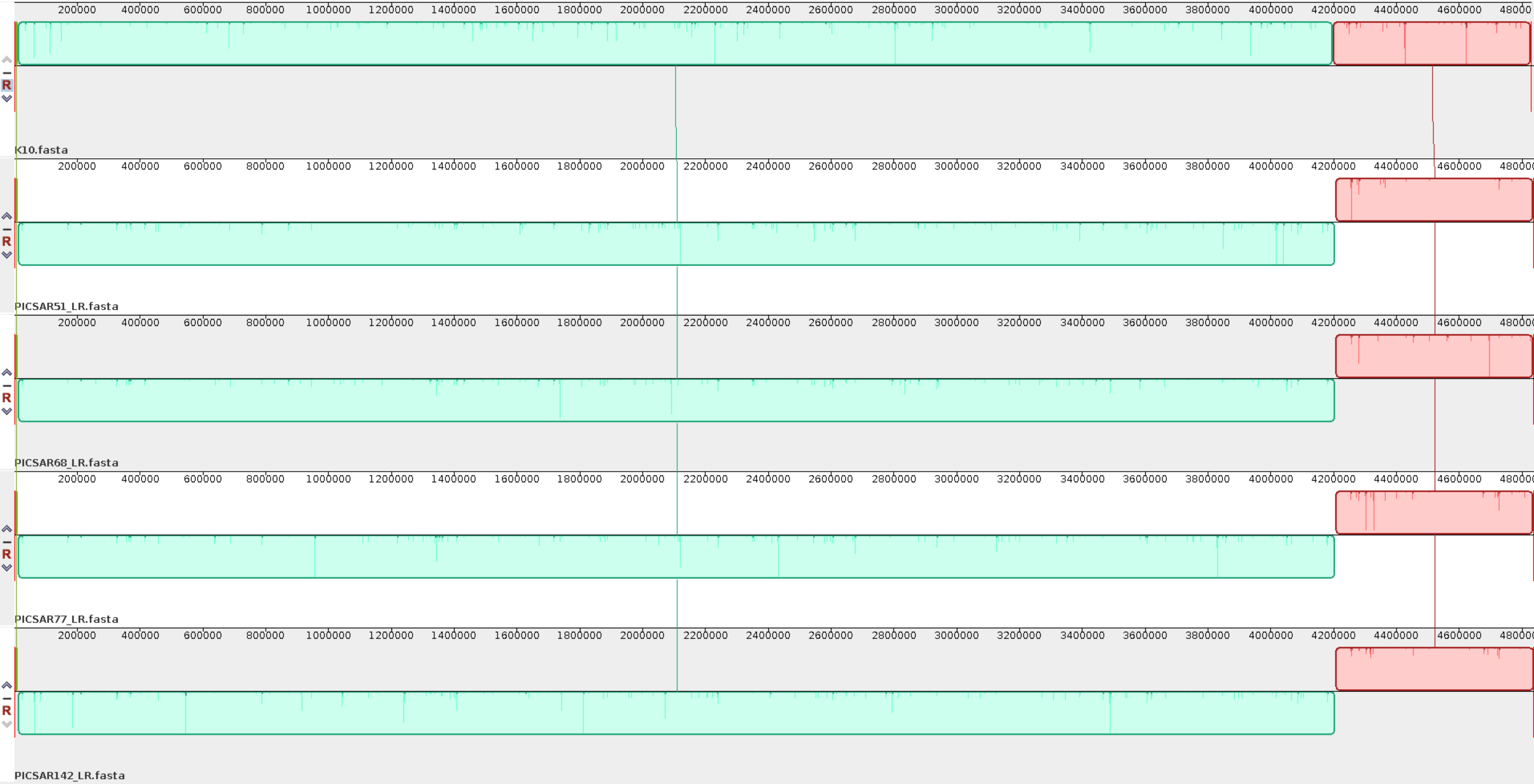
200000 400000 600000 800000 1000000 1200000 1400000 1600000 1800000 2000000 2200000 2400000 2600000 2800000 3000000 3200000 3400000 3600000 3800000 4000000 4200000 4400000 4600000 4800000
K10.fasta
PICSAR51_LR.fasta
PICSAR68_LR.fasta
PICSAR77_LR.fasta
PICSAR142_LR.fasta

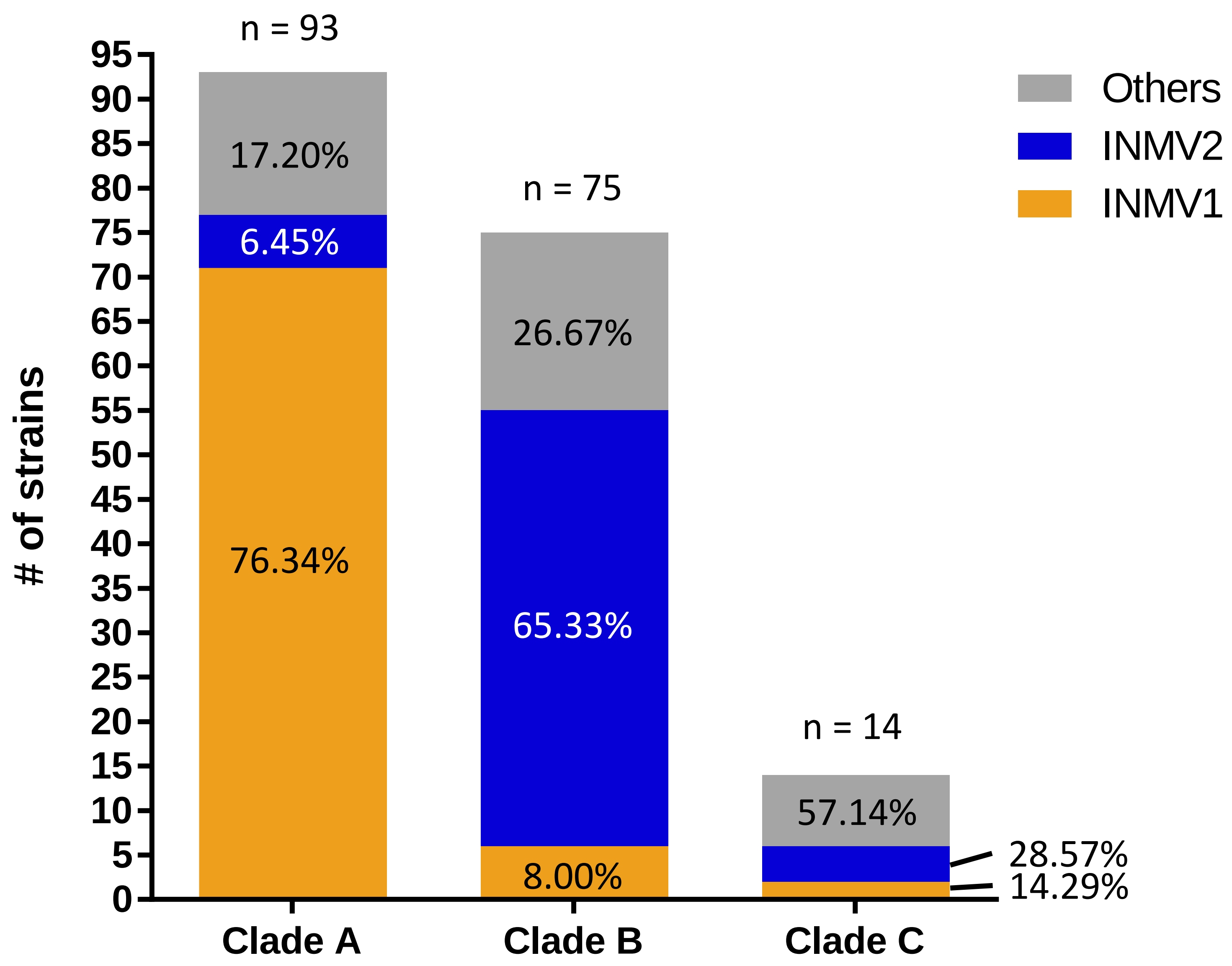
n = 93
n = 75
n = 14
95
90
85
80
75
70
65
60
55
50
45
40
35
30
25
20
15
10
5
0
# of strains
17.20%
6.45%
76.34%
26.67%
65.33%
8.00%
57.14%
28.57%
14.29%
Clade A
Clade B
Clade C
Others
INMV2
INMV1

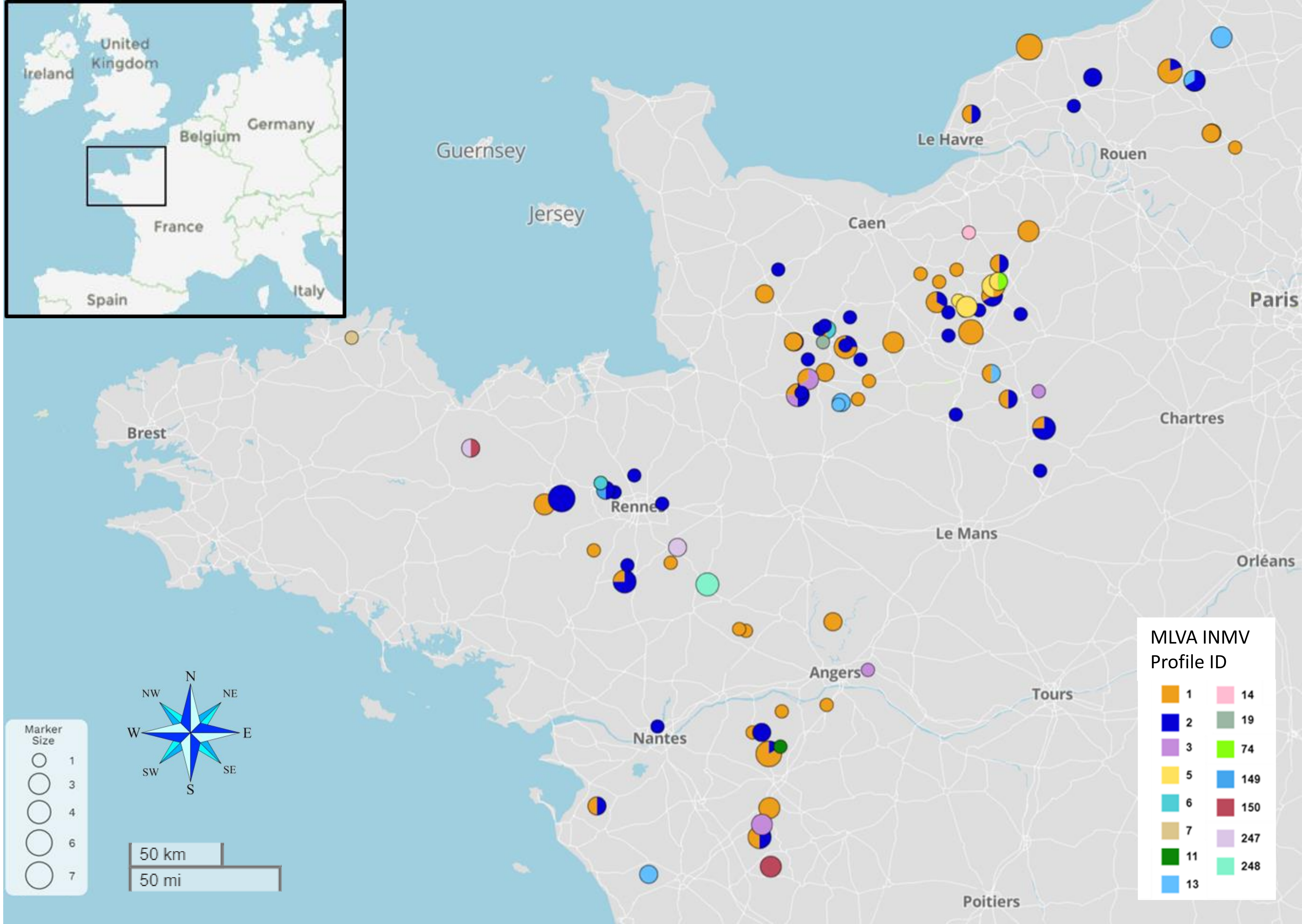
United Kingdom
Ireland
Belgium
Germany
France
Spain
Italy
Guernsey
Jersey
Le Havre
Rouen
Caen
Paris
Chartres
Brest
Renne
Le Mans
Orléans
Angers
Tours
Nantes
Poitiers
MLVA INMV Profile ID
1
2
3
5
6
7
11
13
14
19
74
149
150
247
248
Marker Size
1
3
4
6
7
N
NE
E
SE
S
SW
W
NW
50 km
50 mi
